# Statistical Nonparametric fMRI Maps in the Analysis of Response Inhibition in Abstinent Individuals with History of Alcohol Use Disorder

**DOI:** 10.3390/bs12050121

**Published:** 2022-04-21

**Authors:** Ashwini Kumar Pandey, Babak Assai Ardekani, Kelly Nicole-Helen Byrne, Chella Kamarajan, Jian Zhang, Gayathri Pandey, Jacquelyn Leigh Meyers, Sivan Kinreich, David Balin Chorlian, Weipeng Kuang, Arthur T. Stimus, Bernice Porjesz

**Affiliations:** 1 Henri Begleiter Neurodynamics Laboratory, Department of Psychiatry and Behavioral Sciences, SUNY Downstate Health Sciences University, 450 Clarkson Avenue, MSC #1203, Brooklyn, NY 11203, USA; chella.kamarajan@downstate.edu (C.K.); jian.zhang@downstate.edu (J.Z.); gayathri.pandey@downstate.edu (G.P.); jacquelyn.meyers@downstate.edu (J.L.M.); sivan.kinreich@downstate.edu (S.K.); david.chorlian@downstate.edu (D.B.C.); weipeng.kuang@downstate.edu (W.K.); arthur.stimus@downstate.edu (A.T.S.); bernice.porjesz@downstate.edu (B.P.); 2 Center for Biomedical Imaging and Neuromodulation, The Nathan S. Kline Institute for Psychiatric Research, 140 Old Orangeburg Road, Orangeburg, NY 10962, USA; babak.ardekani@nki.rfmh.org (B.A.A.); kbyrne@exponent.com (K.N.-H.B.)

**Keywords:** executive functions, abstinent AUD, response inhibition, reactive stopping, right hemisphere inhibition network, common cognitive control network mechanisms, functional neuroimaging

## Abstract

Inhibitory impairments may persist after abstinence in individuals with alcohol use disorder (AUD). Using traditional statistical parametric mapping (SPM) fMRI analysis, which requires data to satisfy parametric assumptions often difficult to satisfy in biophysical system as brain, studies have reported equivocal findings on brain areas responsible for response inhibition, and activation abnormalities during inhibition found in AUD persist after abstinence. Research is warranted using newer analysis approaches. fMRI scans were acquired during a Go/NoGo task from 30 abstinent male AUD and 30 healthy control participants with the objectives being (1) to characterize neuronal substrates associated with response inhibition using a rigorous nonparametric permutation-based fMRI analysis and (2) to determine whether these regions were differentially activated between abstinent AUD and control participants. A blood oxygen level dependent contrast analysis showed significant activation in several right cortical regions and deactivation in some left cortical regions during successful inhibition. The largest source of variance in activation level was due to group differences. The findings provide evidence of cortical substrates employed during response inhibition. The largest variance was explained by lower activation in inhibition as well as ventral attentional cortical networks in abstinent individuals with AUD, which were not found to be associated with length of abstinence, age, or impulsiveness.

## 1. Introduction

Inhibition, working memory, and cognitive flexibility are three core dissociable executive function (EF) components [1,2,3]. Higher-order EF skills, such as reasoning, planning, and problem solving are subserved by these EF component processes and are adversely affected by their deficiencies [4,5]. Core EF components are, therefore, not only essential to complete the task at hand but are also important for cognitive, affective, and social development as well as success in school and in life [1]. Evidence suggests that EF components are the first to be adversely affected, often disproportionately, by stress [6,7], sadness [8], loneliness [9], sleep deprivation [10], or poor physical fitness [11]. Their deficits are manifested in various psychiatric and clinical conditions, including alcohol use disorder [12,13,14,15].

Alcohol use disorder (AUD) is a clinical/mental health condition resulting from heavy, chronic, and maladaptive use of alcohol and is characterized by cognitive, affective, and social impairments shown to be related to its adverse effects on brain structure and function [12,13,14,15]. Although some of these impairments may improve with abstinence, indicative of either damage reversal or compensatory brain mechanisms [16], some studies have reported persistent cognitive dysfunction, even after long-term abstinence [16,17]. For example, although EF impairments are reported to lessen with abstinence duration (cross-sectional studies) and demonstrated recovery (longitudinal studies) in AUD [18,19], several studies have also reported persistent EF impairments in patients with AUD, despite long periods of abstinence [16,20,21,22,23,24], rendering the findings equivocal. Therefore, additional studies are needed to confirm the nature and extent of these impairments in abstinent individuals with AUD.

Among the cognitive processes adversely affected by alcohol, impairments in inhibitory processing are thought to be responsible for impulsiveness and disinhibitory behaviors, considered to be the cardinal characteristics of externalizing psychiatric disorders such as AUD [25]. Therefore, the characterization of inhibitory brain deficits that may persist even after the cessation of drinking is crucial to prevent the recurrence of risky drinking behaviors. Although most impulses are affective in nature and acquire appetitive or aversive salience, research into the neural architecture of motor response control, conceptualized as “reactive stopping” and studied using Stop signal and Go/NoGo tasks, has made the greatest advances, as behavior can be better operationalized and the target of the stopping of a motor response can be better understood [26]. Therefore, better characterization of the functional brain substrates involved in motor response inhibition in abstinent AUD would improve the understanding of the nature and extent of persisting inhibitory deficits in abstinent AUD, which could aid in the design of rehabilitation strategies.

Until recently, a dominant view in the functional magnetic resonance imaging (fMRI) literature was the “right frontal” hypothesis, which proposes that response inhibition activates a network of regions within the right frontal brain areas, in which right inferior frontal cortex (rIFC) is a key component (for a review see [26,27,28]). There is growing evidence, however, to support an alternative view proposed by Hampshire that there is no inhibitory module within the rIFC. Instead, response inhibition recruits a “domain general” functionally heterogeneous ensemble of rIFC networks and associated posterior cortical regions, which can be dissociated from each other in the context of other task demands [29,30,31,32,33,34]. Consistent with this alternative view, recent systematic reviews of neuroimaging studies have also indicated the involvement of multiple functional networks in response inhibition and their relative impairments in human drug addiction conditions, including AUD [35]. Proponents of the “right frontal” hypothesis have also advocated for a richer model of inhibition that would also include “proactive” and “selective” along with “reactive” inhibitory control that share network mechanisms with broader cognitive control processes [26,28]. The term “reactive stopping” explains the global inhibitory impacts through the fronto-subthalamic circuitry, a hyperdirect mode of implementing inhibition that is often employed in a reflexive and relatively automatic manner. On the other hand, “proactive” and “selective” inhibition can better explain the frontostriatal inhibitory system, an indirect mode of implementing inhibition [26,28] that is often employed in a voluntary, effortful, and controlled manner. Furthermore, although various tasks used to study inhibition are routinely classified as assessing reactive, proactive, or selective inhibition, in real-world situations, inhibitory functions and tasks often comprise varying degrees of automatic and effortful aspects of inhibition, which may be reflected in relative activation/deactivation patterns within the functional circuitry of the brain, regardless of the tasks used [26,29]. Therefore, understanding brain functional networks of inhibition in a more holistic manner that also considers how common network mechanisms support diverse cognitive processes may be ecologically valid and useful for the design of interventional and rehabilitation strategies to treat clinical conditions that exhibit dysfunctional inhibitory processing.

Most studies that have reported persistent inhibitory impairments despite long periods of abstinence from alcohol have used neuropsychological tests [16,17,18,19,20,21,22,23,24]. However, relatively few fMRI studies have examined response inhibition and executive control networks in abstinent individuals with AUD [36,37]. For example, two fMRI studies that used Go/NoGo tasks to study inhibition [36,37] reported enhanced BOLD activation in abstinent AUD relative to control participants in the fronto-striatal-parietal network [36,37], as well as in several areas involved in visual processing and cognitive and impulse control [36], without impairment in task performance. The authors interpreted these findings as involving a compensatory strategy for impaired cognitive processing. Dresler, et al. [38] found no difference in frontal brain activity during a verbal fluency task between long-term abstinent AUD and control groups, suggesting an increase in frontal brain activity with continued abstinence based on findings of lower frontal brain activity in short-term abstinent AUD compared to controls. On the other hand, Li, et al. [39] reported lower activation of the right dorsolateral prefrontal cortex (DLPFC) and other cortical and subcortical brain structures during a stop signal task. Akine, et al. [40] reported lower activity in the right DLPFC and anterior cingulate cortex (ACC) in abstinent individuals with AUD compared to controls during a recognition task. Furthermore, in a review of resting-state and event-related fMRI studies, Fein and Cardenas [41] argued that abstinence (short-term) and its maintenance (long-term) are associated with compensatory changes in synchrony, so that the “top-down” executive control network has greater synchrony and the “bottom-up” stimulus-driven appetitive drive network has reduced synchrony compared to controls. These studies suggest that results regarding brain activity in executive inhibitory control regions and networks for various lengths of abstinence are inconsistent [41,42]. Therefore, an apparent disconnect appears to be present between neuropsychological and neuroimaging study findings, where most neuropsychological studies have suggested the presence of inhibitory impairments despite long periods of abstinence, while neuroimaging results have been inconsistent regarding brain activation, often in the absence of impaired task performance.

Most fMRI studies have used Statistical Parametric Mapping (SPM) for Blood-Oxygen-Level-Dependent (BOLD) analysis. SPM requires data to fulfil parametric assumptions, which often is difficult to achieve in biophysical systems such as the brain [43] and may also contribute towards inconsistencies in the results observed. Studies have shown that depending on the repetition time, paradigm, and parameter settings, parametric significance thresholds in SPM can either be either conservative or liberal compared to those of nonparametric random permutation tests. The main reason for this high variability seems to be that the global auto correlation correction in SPM fails to model the spectra of residuals, especially for short repetition times [44]. On the other hand, the nonparametric fMRI analysis offers the freedom to use a test statistic to compare experimental conditions. This robustly corrects for multiple comparisons and allows the incorporation of biophysically motivated constraints in the test statistic, which may drastically increase the sensitivity of the statistical test [43,45].

It is, therefore, clear that literature involving brain regions responsible for different types of inhibition, the adverse effects of heavy and maladaptive alcohol use on inhibitory processes, and associated brain regions are challenged by myriad complexities, ranging from the effects of alcohol, its quantity, clinical conditions, course of illness, and recovery, on one hand, and methodological differences and analytical limitations on the other, e.g., [16]. While inhibitory deficits that occur in different stages of alcohol use have been studied previously [13,14,15], due to differences in the methodologies and outcome measures used, extant results are inconsistent. Further, although neuroimaging studies have examined response inhibition in active AUD, very few fMRI studies have examined it in abstinent individuals with AUD. Therefore, more research is needed to assess the involvement of brain regions and their network that are consistently activated during response inhibition to understand the extent and nature of inhibitory abnormalities that persist after the cessation of drinking. The present study aims to examine performance during a Go/NoGo task with the objectives being (1) to characterize the neuronal substrates associated with response inhibition using a rigorous permutation-based fMRI analysis that controls for false positive rates to determine regions that are consistently activated across participants and (2) to determine whether these regions are differentially activated between abstinent AUD and control groups.

## 2. Materials and Methods

### 2.1. Participants

Based on the inclusion criteria, the sample comprised 60 male participants, including 30 abstinent AUD individuals who met the DSM-IV criteria for Alcohol Dependence (M_age_ = 41.42 years, SD = 7.31) and 30 healthy controls (M_age_ = 27.44 years, SD = 4.74). Recent studies on the same sample can be referred to for further details [24,46,47]. Relevant details pertaining to the fMRI during the Go/NoGo task performance is included here. Controls were recruited through advertisements and screened to exclude those with a history of major medical, psychiatric, or substance-related disorders. The participants with AUD were recruited from alcoholism treatment centers in and around New York City after they had been detoxified and were not in withdrawal. Participants had a diagnosis of AUD, were currently abstinent, and did not meet the DSM-IV criteria for other psychiatric and substance use disorders (see Table 1). Additional exclusion criteria were hearing/visual impairment, liver disease, a history of head injury, or moderate to severe cognitive deficits, as indicated by a Mini Mental Status Examination (MMSE) score <21 [48].

A modified version of the semi-structured assessment for the genetics of alcoholism (SSAGA), the polydiagnostic clinical interview, was administered to assess alcohol/substance use and related disorders [49]. Participants were instructed to abstain from alcohol and other substances for at least 5 days prior to completing the assessments and neuroimaging and were screened on the day of testing using an alcohol breathalyzer test. Individuals who tested positive or reported substance use were either rescheduled or excluded from the study. The Barrett Impulsiveness Scale (BIS-11) was administered to identify common impulsive and nonimpulsive behaviors and preferences in the study participants [50]. Standard protocols of recruitment for MRI were followed to ensure participants’ safety during the scan. A written informed consent was obtained from each participant. Experimental procedures and human research protection plans were carried out in accordance with the Declaration of Helsinki and were approved by the Institutional Review Boards of the SUNY Downstate Health Sciences University and The Nathan S. Kline Institute for Psychiatric Research (NKIPR).

### 2.2. Go/NoGo Task

**Figure 1:**
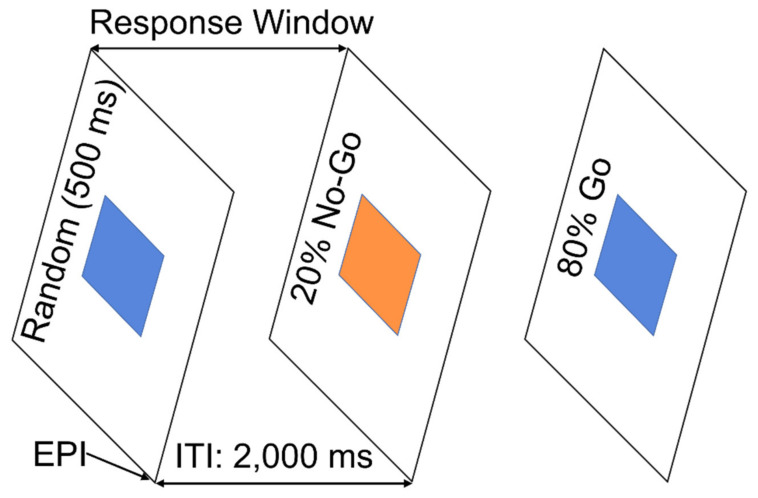
Schematic diagram illustrating the Go/NoGo task.

In this task, 200 visual stimuli, either a blue or an orange square, were presented to the participants for a duration of 500 ms at regular 2000 ms intervals. Participants were asked to press a button as quickly as possible with their right index finger if they saw a blue square (a Go trial) and refrain from pressing the button if they saw an orange square (a NoGo trial). Both speed and accuracy of performance were emphasized. There were 160 (80%) Go trials and 40 (20%) NoGo trials in the task. The beginning of each trial was synchronized to the beginning of an Echo Planer imaging (EPI) volume acquisition by a trigger pulse sent from the MRI scanner to the stimulus presentation software. The 200 Go and NoGo trials were randomly interspersed without any constraints over the course of the 200 EPI acquisitions (400 s). The response/refrain window was 2000 ms, during which the Reaction time (RT) to the successful Go trials was recorded, whereas responses to NoGo trials in this time window were identified and recoded as errors. Performance accuracy measures were derived by calculating the average number of correct responses for Go and NoGo trials separately. Please see the illustration below in Figure 1.

### 2.3. Image Acquisition

Imaging was performed at the NKIPR using the Siemens MAGNETOM Tim/Trio, a 3 Tesla MRI scanner (Erlangen, Germany). Blood oxygenation level dependent (BOLD) fMRI scans were acquired using a T2*-weighted gradient echo single-shot EPI sequence with TR = 2000 ms, TE = 30 ms, flip angle = 80°, FOV = 240×240 mm^2^, and matrix size = 96×96. A total of n=200 EPI volumes were obtained from each participant. Each volume covered nearly the whole brain and consisted of 36 transverse slices of 2.8 mm thickness and a 0.7 mm gap, yielding a voxel size of 2.5×2.5×3.5 mm^3^. Each EPI acquisition triggered a Go/NoGo trial by sending a trigger pulse to the stimulus presentation software. In addition to the EPI sequence, we acquired a high-resolution three-dimensional T1-weighted magnetization-prepared rapid gradient-echo (MPRAGE) volume with TR = 2500 ms, TE = 3.5 ms, TI = 1200 ms, flip angle = 8°, matrix size = 256×256×192, and voxel size = 1×1×1 mm^3^; and a turbo spin-echo (TSE) proton-density (PD)-weighted volume with TR = 7000 ms, TE = 11 ms, TI = 1200 ms, matrix size = 256×256×72, and voxel size = 1×1×2 mm^3^. The MPRAGE and PD volumes were used for intersubject registration and EPI distortion correction purposes, as described below.

### 2.4. fMRI Pre-Processing

The *3dvolreg* module of the Analysis of Functional NeuroImages (AFNI) software package [51] was used to detect and correct participants’ motion in the fMRI sequence. The accuracy of this motion detection method was validated in a separate study [52]. The procedure yielded a motion-corrected 4D image sequence of size 96×96×96×200 as well as 6 estimated rigid-body motion time series (3 translations and 3 rotations). These time series were used as nuisance covariates in the general linear model (GLM) used for activation detection, as described below. The motion parameters did not differ statistically between groups.

The 200 motion-corrected EPI volumes were averaged. An intensity threshold was applied to the average volume to obtain a brain mask for each participant. Subsequent voxel-wise analyses were confined to the brain mask voxels. A principal component analysis (PCA) was performed on the motion-corrected fMRI sequence. The first principal component capturing the greatest amount of variance in the data was also used as a nuisance covariate in the GLM.

### 2.5. Subject-Level BOLD Response: NoGo vs. Go

For each of the 60 participants in our study, we separately performed voxelwise fitting of the BOLD signal to a GLM:

(1) $$y_{v}=X\beta_{v}+e_{v}$$

In this model, $y_{v}$ is the n×1 vector of BOLD signal observations after motion-correction at voxel v (n=200); X is the n×p  design matrix described below; $\beta_{v}$ is the p×1 vector of unknown parameters; and $e_{v}$ is an n×1 vector of random noise.

In our analysis, the number of columns in the design matrix was p=11. The first column was the eigenvector corresponding to the largest eigenvalue obtained by the PCA of the motion-corrected fMRI sequence across all brain voxels. As mentioned in the Section 2.4, this was included as a nuisance covariate. The next six columns of X were occupied by the 6 estimated rigid-body motion parameters obtained using AFNI’s *3dvolreg*. As mentioned in the Section 2.4, these were also included in the design matrix as nuisance covariates. Finally, columns 8–11 of X were designed to model the hemodynamic response during the Go/NoGo task, as described in the following paragraph.

During the performance of the Go/NoGo task, four types of mutually exclusive events can occur: (1) correct Go events in which the participant pushes the button after a Go stimulus; (2) correct NoGo events in which the participant withholds their response to a NoGo stimulus; (3) errors of omission in which the participant fails to push the button during a Go trial; and (4) errors of commission in which the participant fails to inhibit themselves from pushing the button during a NoGo trial. Thus, the performance of the participant can be summarized by an n×4 binary matrix $Z=\left[z_{1}\;z_{2}\;z_{3}\;z_{4}\right]$, where a value of 1 in a given row indicates the occurrence of 1 of the 4 events defined above. Since these events are mutually exclusive, each row of matrix Z can only have one nonzero entry. We modeled the hemodynamic response to each event type by the convolution of $z_{j}$ (j=1,2,3,4) with a canonical gamma hemodynamic response function (HRF), as described by Ardekani, et al. [53] and Rangaswamy, et al. [54]. The model thus obtained comprised the last 4 columns of X. Specifically, if we represent the HRF by h, the last 4 columns of X are given by $h*z_{j}$ where ∗ denotes the convolution operation. Alternatively, we can represent the n×4 submatrix comprised of the last 4 columns of the design matrix as h∗Z, where it is understood that the convolution operation is applied to the columns of Z.

Let G represent the pseudo-inverse of the p×p  matrix $X^{t}X$. The parameters of the linear model are estimated as ${\hat{\beta }}_{v}=GX^{t}y_{v}$. Since we were interested in the brain networks that are activated during successful inhibition of a motor response to a visual stimulus, we looked for voxels for which the contrast $C^{t}{\hat{\beta }}_{v}$ was large relative to the amplitude of the random variations, where $C^{t}={\left[0\;\cdots -1\;1\;0\;0\right]}^{t}$. A variable that captures this notion is given by

(2) $$t_{v}=\frac{C^{t}{\hat{\beta }}_{v}}{\sqrt{{\hat{\sigma }}^{2}C^{t}GC}}$$

where ${\hat{\sigma }}^{2}=\left(y^{t}y-y^{t}X^{t}GXy\right)/df$ and $df=n-1-rank\left(G\right)$.

Note that under certain normality and independence conditions, $t_{v}$ would have a Student’s *t* distribution with df degrees-of-freedom. However, we did not make such assumptions and simply regarded $t_{v}$ as a variable designed to indicate the level of activation at voxel v. The statistical significance of $t_{v}$ values was determined based on the nonparametric permutation-based methods described in Section 2.8. By computing this index for all voxels, we obtained a subject-level BOLD response map for each of the 60 participants in our study.

### 2.6. Intersubject Registration

The processing described in the Section 2.5 resulted in 60 BOLD response maps, one for each participant. These activation maps were in the native space of the participants’ fMRI acquisition. Since our objective was to obtain an activation map for the entire group (see below), these maps had to be registered into a common space. This was performed using automatic registration toolbox (ART) software (www.nitrc.org/projects/art, accessed on 11 March 2022). The MPRAGE images were skull-stripped using the *brainwash* module in ART. These images were nonlinearly registered to the *Colin27* [55] image in the MNI space using ART’s *3dwarper* module [56]. The MPRAGE and PD images were registered together using ART’s *rigid-body registration* module [57]. For distortion correction, the PD and the average of the motion-corrected fMRI images were nonlinearly registered using ART’s *unwarp2d* module. The three registration transformations (fMRI-to-PD, PD-to-MPRAGE, and MPRAGE-to-MNI) were combined numerically to obtain a fMRI-to-MNI transformation and applied to the participants’ activation maps to transform them to MNI space using trilinear interpolation.

### 2.7. Group-Level BOLD Response

Once all 60 subject-level BOLD response maps had been transformed to the common MNI space, they were smoothed with a 3D isotropic Gaussian smoothing kernel with a 3 mm standard deviation (SD), and the median image was computed to represent a BOLD response map for the entire group. The median image was chosen in place of the mean image to reduce the effect of outliers.

### 2.8. Differential BOLD Response: NoGo vs. Go

In the group-level BOLD response map obtained as the median of the 60 spatially normalized subject-level BOLD response maps, voxels with large absolute values represented potentially significant BOLD responses to the fMRI task. Thresholding was necessary to separate the voxels (if any) from statistically significant BOLD responses. As threshold levels based on assumptions about the spatial distribution of the group-level BOLD response may not necessarily hold and may lead to errors [39], we used a nonparametric permutation-based statistical analysis technique to identify the regions in the brain with significant BOLD responses to successful NoGo events. In this procedure, we repeated the computations described in the Section 2.5, Section 2.6 and Section 2.7 1000 times; that is, (1) computation of the subject-level BOLD response maps for each of the 60 participants; (2) nonlinear transformation of the individual maps to the MNI space; and (3) Gaussian smoothing and computation of the median image as the group-level BOLD response map. However, for each of the 1000 runs, in step (1), we randomly permutated the rows of the performance matrix Z. Therefore, during these runs, the assumed behavior of the participants in the scanner did not match their actual behavior, although the level of their performance (e.g., accuracy) was preserved.

Thus, we obtained 1000 group-level BOLD maps. We noted the maximum and minimum values of each group-level BOLD map and computed two empirical distributions: one for the maxima (with positive values) and one for the minima (with negative values). Using these data, we defined 6 threshold levels: $T_{0.001}^{U}$, $T_{0.01}^{U}$, $T_{0.05}^{U}$, $-T_{0.001}^{L}$, $-T_{0.01}^{L}$, and $-T_{0.05}^{L}$. These values were then used to threshold the one true group-level BOLD map that had been obtained using the participants’ actual Z performance matrix. For example, under the null hypothesis that there are no voxels anywhere in the brain with a significantly negative BOLD response, the empirical probability that a voxel intensity level anywhere in the group-level BOLD map is below $-T_{0.001}^{L}$ is less than 0.001. Similarly, as another example, under the null hypothesis that there are no voxels anywhere in the brain with a significantly positive BOLD response, the empirical probability that a voxel intensity anywhere in the group-level BOLD response map is above $T_{0.01}^{U}$ is less than 0.01.

In summary, we conducted a total of 1001 analyses and computed 60,060 subject-level BOLD response maps, nonlinear transformations, and smoothing and median operations. In the first 1000 analyses, the performance matrix Z, while realistic, did not bear any resemblance to the actual behavior of the participants’ performing the Go/NoGo task. Only in the last analysis did the timing of events match the participants’ actual performance. We then thresholded the final true map based on the frequency of the observed maxima and minima values under the null hypothesis. This procedure controls for the family-wise error rate (FWER). We conservatively only chose contiguous voxel clusters (size greater than 100 mm^3^) that were significant after correcting for FWER.

### 2.9. Differential BOLD Response: AUD vs. Controls

Using the threshold values obtained based on the permutation analysis, we considered voxels with response levels greater than $T_{0.05}^{U}$ as having a significantly positive BOLD response and those with levels less than $-T_{0.05}^{L}$ as having a significantly negative BOLD response at the α=0.05 level while controlling for FWER as a result of successful inhibition (NoGo event) by the participants. These thresholds yielded a total of p=35,992 voxels with significant positive/negative responses. Thus, we represented the entire BOLD response data with a n×p matrix with X elements $x_{ij}$ representing the response level in voxel j of participant i, where $\left(i=1,2,\cdots ,60\right)$ and (j=1,2,⋯,35,992). We then approximated matrix X by a singular value decomposition as $\tilde{X}=UDV^{T}$, where U is an n×K matrix with columns $u_{k}$, *D* is a K×K diagonal matrix with elements $d_{k}$, V is a p×K matrix with columns $v_{k}$, and K is the rank of the approximation. It is well known that $\tilde{X}$ results in the minimum Frobenious norm ${||X,-,\tilde{X}||}_{F}$ amongst all rank K matrices.

The $v_{k}$ $\left(k=1,2,\cdots ,K\right)$ are known as the principal components (PC) of data matrix X or, in this case, can be regarded as eigenimages, since they originate from an imaging experiment. The $u_{k}$ represent the PC scores of each of the n participants with respect to the eigenimage $v_{k}$. They can also be thought of as coordinates of the projection of data matrix X in the direction of vector $v_{k}$ that are normalized to have a mean of zero and a variance of 1. The scalar values $d_{k}$ are arranged in descending order and represent the standard deviation of the projection points of matrix X in the direction of vector $v_{k}$.

If there were significant differences between the BOLD responses of the AUD and control groups, we would expect that variation would be captured in one of the top K components $v_{k}$, and the PC scores $u_{k}$ would be significantly different between AUD and control groups. Thus, we examined this question to determine whether group differences existed between the AUD and control groups in the level of BOLD responses to successful NoGo trials. Nonparametric Mann–Whitney *U* Tests were performed on ten PC scores $\left(u_{1-10}\right)$ to explore differences between the control and AUD groups in the level of BOLD responses to successful NoGo trials. A sparse PCA [58] was performed to reduce the nonzero elements of $v_{k}$.

### 2.10. Analyses of Demographic, Clinical, and Performance Data

Student’s *t*-tests were computed to determine group differences in age, education, alcohol/substance use variables, Go/NoGo behavioral performance accuracy, average reaction times, and BIS-11 scores. Pearson’s correlation (*r*) was performed between age and the PC scores ($u_{k}$), between age and performance accuracy, between age and average reaction time, between BIS-11 scores and BOLD activation outcome variables, and between length of abstinence and the BOLD activation outcome variables of the participants. The values were adjusted for multiple correction using the Bonferroni method. The α significance was set at less than the 0.05 level.

## 3. Results

### 3.1. Demographic, Clinical, and Performance Data

Data presented in this study is included as a Supplementary Materials. Statistical descriptions, tests results, and respective *p*-values for the sociodemographic, clinical, and performance data are presented in Table 1. Participants in the AUD group were significantly older, had fewer years of educational attainment, had an earlier age of onset of regular drinking, used higher quantities (drinks/day) of alcohol, and had higher frequencies (drinking days/month) of alcohol use during heavy-use periods, longer durations of abstinence, higher quantities of tobacco use, and higher frequencies of marijuana use. However, the groups did not significantly differ in terms of the quantity of occasional alcohol use and the frequency of alcohol and tobacco use during the last 6 months before participating in the study (Table 1). Further, significantly lower performance accuracy levels in the Go trials with slower average reaction times were found in abstinent AUD participants compared to controls, whereas groups did not differ significantly in performance in the NoGo trials. The abstinent AUD had significantly higher levels of impulsiveness on all indices namely, nonplanning, motor impulsiveness, attentional impulsiveness, and total impulsiveness. Age was positively associated with the average reaction time [*r*(58) = 0.41, *p* < 0.001] and negatively associated with the Go performance accuracy [*r*(58) = −0.32, *p* < 0.012] but not significantly correlated with the NoGo performance accuracy [*r*(58) = 0.05, *p* < 0.731] when the entire sample was assessed. However, none of the correlations with age were found to be significant for either group separately, except for average reaction time of the controls [*r*(28) = 0.36, *p* < 0.05]. BIS-11 impulsiveness scores and length of abstinence were not found to be significantly correlated with any of the BOLD activation outcome variables and, therefore, are not reported.

### 3.2. Differential BOLD Response: NoGo vs. Go

**Figure 2:**
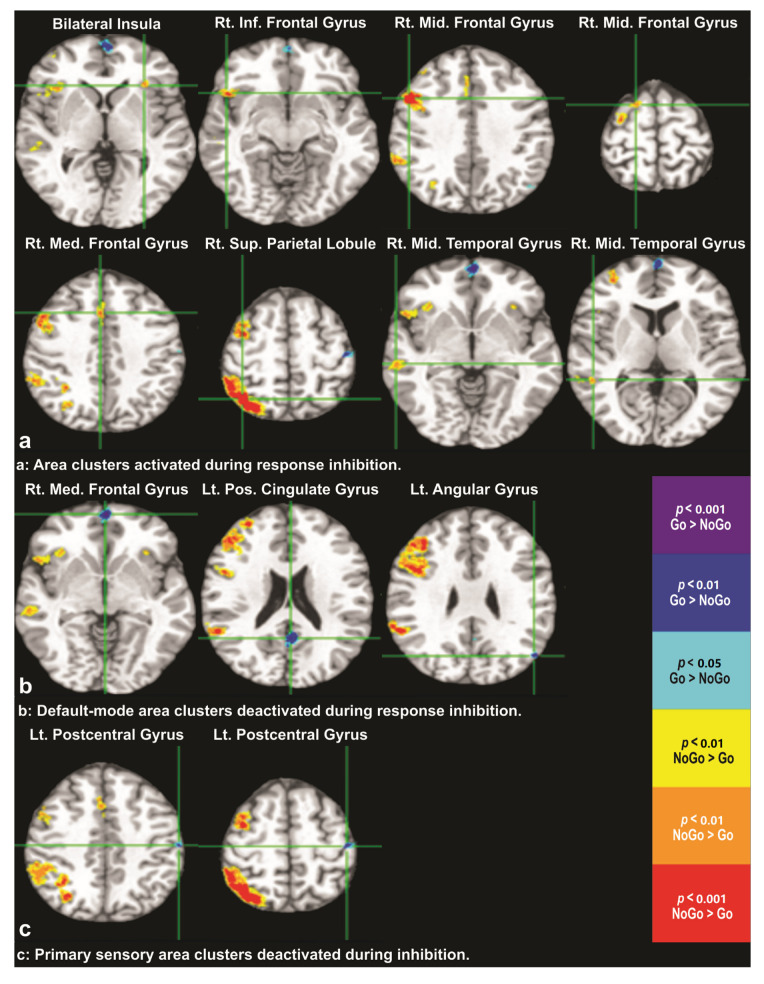
Thirteen voxel clusters where (**a**) 7 regions in the right hemisphere and bilateral anterior insula were activated, (**b**) 3 regions in the default-mode network were deactivated, and (**c**) 2 regions in the primary sensory areas were deactivated during successful response inhibition. All figures are in radiological orientations.

Thresholding the group-level BOLD response map at levels $T_{0.05}^{U}$ and $-T_{0.05}^{L}$ resulted in 13 clusters of voxels (size greater than 100 mm^3^; a total of 35,992 voxels), as shown in Figure 2. Positively valued voxels for which the intensities were above $T_{0.05}^{U}$, $T_{0.01}^{U}$, and $T_{0.001}^{U}$ are shown in yellow, orange, and red, whereas negatively valued voxels for which the intensities were below $-T_{0.05}^{L}$, $-T_{0.01}^{L}$, and $-T_{0.001}^{L}$ are shown in cyan, blue, and purple, respectively and correspond to FWER of *p* < 0.05, *p* < 0.01, and *p* < 0.001. The cluster signs and sizes, MNI coordinates of peaks/troughs, and corresponding brain regions are given in Table 2 and are, thus, not described in the text to avoid redundancy and space constraints. Out of a total of 13 significant clusters, 8 showed positive and 5 showed negative BOLD responses, signifying the activation and deactivation of brain regions within these clusters during successful inhibition, respectively (refer to Figure 1, Table 2).

### 3.3. Differential BOLD Response: AUD vs. Controls

**Figure 3:**
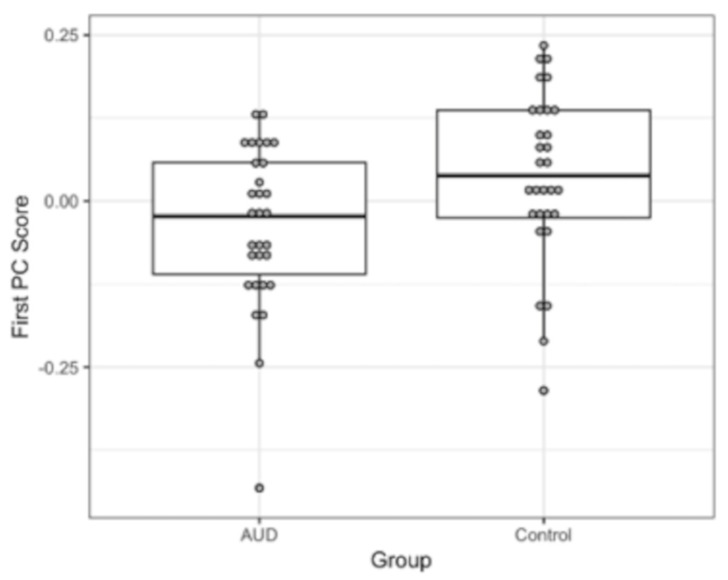
Boxplots of the 1st principal component scores in the AUD and control groups. The scores were significantly different (*p* < 0.02) between groups, as determined by a nonparametric Mann–Whitney U test.

**Figure 4:**
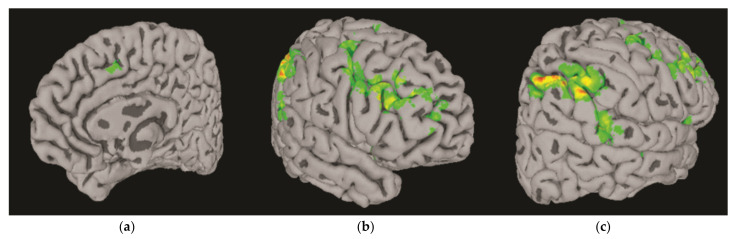
Right hemispheric cortical regions where positive BOLD response in the AUD group was signif-icantly lower relative to in the control group: (**a**) medially presupplementary motor area, (**b**) bi-lateral anterior insula, inferior and middle frontal cortices, and (**c**) posteriorly superior parietal lobule, angular gyri, and middle temporal cortices.

The direction of the first eigenimage $v_{1}$ accounted for 19.6% of the total variance in the total of 35,992 voxels. A Mann–Whitney nonparametric *U* test showed that the first PC scores ($u_{1}$) with respect to this direction were significantly different between the abstinent AUD and control groups (*U* = 285, *p* < 0.02), whereas examining the next nine PC scores $\left(u_{2-10}\right)$ did not reveal any other directions that significantly separated the abstinent AUD and control groups beyond chance. Thus, the largest source of variance in the pattern of NoGo BOLD response in our data was, in fact, mainly due to the differential activation between the abstinent AUD and control groups. Figure 3 shows boxplots of the $u_{1}$ scores, which illustrate lower PC scores for the abstinent AUD group compared with the control group. This can be considered an omnibus statistical test for the null hypothesis that there are no differences in the BOLD responses between the abstinent AUD and control groups. A disadvantage of this technique is that it has weak localization power; that is, all 35,992 voxels with a significant BOLD response (Figure 2) contributed to $v_{1}$. It is possible that the differential BOLD responses are contained in a subset of these voxels. Thus, a threshold seems necessary to further localize the voxels that contributed to the differential BOLD responses to the NoGo condition between the abstinent AUD and control groups. For this purpose, we applied the method of sparse PCA [58]. In this approach, in addition to the usual PCA constraints on $v_{1}$ and $u_{1}$, to have a norm of 1, an $l_{1}$ norm constraint is imposed on $v_{1}$ such that ${||v_{1}||}_{1}\leq c$, where c is a sparsity parameter. The $l_{1}$ norm constraint acts as a threshold to force “weak” elements of $v_{1}$ to zero. Using this approach, we reduced the number of nonzero elements of the first PC by 55% to 15,922 voxels while keeping the effect size of the difference in PC scores between controls and abstinent AUD participants at approximately the same level. Figure 4 shows the brain regions in $v_{1}$ in which the collective BOLD response in controls was significantly higher than in abstinent AUD in the bilateral anterior insula, right inferior, middle, and medial frontal gyri, which include the presupplementary motor area (pre-SMA) and paracingulate cortical regions, the right parietal superior parietal lobule, the angular gyrus, and the middle temporal gyri. The correlation between the first principal component score ($u_{1}$) and age was found to be nonsignificant [*r*(58) = 0.20, *p* < 0.132], suggesting age did not contribute to the variance explained by the first PC score ($u_{1}$).

## 4. Discussion

The main findings of the present study are as follows: (1) the nonparametric fMRI analysis of BOLD responses across both abstinent AUD and control groups on successful NoGo trials showed cortical activation in the bilateral anterior insula, right inferior, middle, and medial frontal gyri, which include the pre-SMA and paracingulate cortical regions, the right superior parietal lobule, and the middle temporal gyri (Figure 2a); (2) the BOLD response also showed cortical deactivation in the right medial frontal, left posterior cingulate, angular gyri (Figure 2b), and postcentral regions (Figure 2c); (3) Mann–Whitney nonparametric U tests showed that the first PC score ($u_{1}$) that explained variance was due to a difference between groups where, compared to controls, the abstinent AUD group showed lower cortical activation during successful response inhibition in the right pre-SMA and paracingulate regions (Figure 4a), inferior and middle frontal regions (Figure 4b), superior and inferior parietal lobules, angular gyri, and middle temporal regions (Figure 4c). Age was not significantly associated with the first PC score ($u_{1}$), which captured the variance due to differential levels of activation between the abstinent AUD and control groups; (4) abstinent AUD group had longer average reaction times and performed less accurately on Go trials but not on NoGo trials. Age was negatively associated with Go performance accuracy and positively associated with the average reaction time; (5) the abstinent AUD group scored more highly on all impulsiveness indices compared to the control group; and (6) impulsiveness and length of abstinence were not significantly associated with the BOLD activation outcome variables. The findings are discussed below in light of current neuroimaging literature on response inhibition and its impairment in AUD.

### 4.1. Inhibitory Functions and Its Brain Substrates

Based on the results of fMRI studies examining neural correlates of response inhibition conducted in the last two decades, several models of inhibitory control have been proposed (for a review see [29,35]). Among them, arguably the most prominent and influential models are those proposed by Aron and Hampshire. Aron and colleagues proposed that response inhibition activates a module within the right frontal brain areas, in which the inferior frontal cortex (rIFC) is the key component [26,27,28]. Aron [26] delineated a neural system for “reactive inhibitory control” that involves stopping a response that includes the rIFC, dorsomedial frontal cortex, especially the pre-SMA region, and the subthalamic nucleus (STN). The rIFC plays a role in attentional detection involving the dorsal rIFC region known as the inferior frontal junction (IFJ), whereas the ventral rIFC region implements inhibitory control. The pre-SMA region is implicated in the preparation and selection of a response, whereas the STN inhibits the basal ganglia output and the motor system generally [26].

Hampshire and colleagues, on the other hand, proposed that there are no inhibitory modules in the frontal lobe. They presented response inhibition as an example of a broader class of control processes [29,30,31,32,33]. Neural areas associated with specific inhibitory control tasks coexist as common network mechanisms, and behavioral inhibition is an emergent property of spatially distributed functional networks, each of which supports diverse cognitive processes. These control processes are supported by the same set of fronto-parietal networks that exert control by modulating local lateral inhibition processes, which occur ubiquitously throughout the cortex [31]. Thus, instead of focusing on how a specific brain region and its connection pathways may support response inhibition, understanding the neural basis of behavioral control may require a more holistic approach that considers how common network mechanisms support diverse cognitive processes [30,32,59].

In the context of these hypotheses of inhibitory functions and their brain substrates, the NoGo activation findings across groups in the present study tend to conform more to the common network mechanism model proposed by Hampshire and colleagues. While findings of predominantly right frontal brain area activation (first 5 images in Figure 2a) suggest the importance of right prefrontal cortical regions in response inhibition circuitry, as proposed in the “right frontal” hypothesis [26,27,28], findings of right fronto-parieto-temporal and ventral attentional network activation (Figure 2a; positive activation direction in Table 2) support the common network mechanisms hypothesis [29,30,31,32]. In addition, we found deactivation in different brain areas during successful response inhibition which, based on their anatomical locations, have been identified as part of the default-mode network (Figure 2b) and motor response regions, which may be related to withholding from the button press response (Figure 2c). Furthermore, deactivation of these regions in the respective functional circuits may serve as validation for the activation network during successful response inhibition (Figure 2a).

Consistent with this, studies have previously reported common regions activated by response inhibition tasks, including the fronto-parietal network, specifically the IFC, pre-SMA, and bilateral parietal regions [60,61], whereas unique neural networks for the three subcategories of processes underlying response inhibition have also been suggested [60,62,63,64,65]. In a recent systematic review of neuroimaging studies of response inhibition and its impairments in human drug addiction, Zilverstand, Huang, Alia-Klein and Goldstein [35] found coactivation of executive control (DLPFC/VLPFC), salience (dorsal ACC, anterior insula, and IPL), and memory (hippocampus/parahippocampus) networks for inhibitory control and their impairments in drug addiction. In a meta-analytic review, Zhang, Geng and Lee [29] studied the potential common and distinct neural substrates of different response inhibition tasks and examined three subcategories of the cognitive processes underpinning response inhibition, namely interference resolution, response withholding, and response cancellation. The authors concluded that independent of the task type, activation of the right hemispheric regions (the IFC, insula, median cingulate, and paracingulate gyri) and the superior parietal cortex is common across the cognitive processes studied. Mapping the activation patterns to a brain functional network atlas revealed that the frontoparietal and ventral attention networks are the core neural systems that are commonly engaged during the different processes of response inhibition. Subtraction analyses elucidated the distinct neural substrates of interference resolution, action withholding, and action cancellation and revealed stronger activation in the ventral attention network for interference resolution than action inhibition.

Furthermore, in credence to the “common network mechanism” hypothesis, proponents of the “right frontal” hypothesis have also advocated for a richer model of inhibition that would also include “proactive” and “selective” along with “reactive” inhibitory control. These types of inhibition share network mechanisms with broader cognitive control processes [26,28]. The term “reactive stopping” explains global inhibitory impacts through the fronto-subthalamic circuitry, a hyperdirect mode of implementing inhibition that is often employed in a reflexive and relatively automatic manner. On the other hand, “proactive” and “selective” inhibition can better explain the frontostriatal inhibitory system, an indirect mode of implementing inhibition [26,28] that is often employed in a voluntary, effortful, and controlled manner. Importantly, although various tasks to study inhibition are routinely classified as assessing reactive, proactive, or selective inhibition, in real-world situations, inhibitory functions and tasks often comprise varying degrees of automatic and effortful aspects of inhibition, which may be reflected in relative activation/deactivation patterns within the functional circuitry of the brain, regardless of the tasks used [26,29]. These findings as well as the results of the present study, suggest that response inhibition is not a unidimensional construct but consists of subcategories of cognitive processes that engage common as well as distinct neural networks.

### 4.2. Impairments of Response Inhibition in Abstinent AUD

Several fMRI studies have examined inhibitory impairments related to alcohol in different groups: in individuals with a current diagnosis of AUD and drug addiction [35,41,66,67,68,69,70], in young nondependent social drinkers with higher levels of use [71], in individuals with a family history of AUD [72], and in terms of the effects of [73] and prenatal exposure [74] to heavy use of alcohol. These studies have reported neural brain activation deficits and impairments in different brain regions and functional networks (see for a review 35). Out of the 30 systematically reviewed studies on drug addiction, including studies on active AUD), Zilverstand, Huang, Alia-Klein and Goldstein [35] noted that 77% of studies consistently reported lower levels of activation in the salience network (anterior insula, dorsal ACC, and IPL), often accompanied by lower activation of executive (DLPFC/VLPFC) and memory (hippocampus/parahippocampus) networks during successful stops [36,69,75,76,77,78,79,80,81] during successful stops [75]. This overall pattern was found to be similar across different types of drug addiction, including alcohol, except for in highly educated ecstasy users, indicating a preserved compensatory response [82]. Therefore, relatively more studies involving active drug and alcohol addiction appear to indicate impairments in redirecting attentional resources, response inhibition, and learning during response inhibition [35].

However, the neuroimaging literature is inconsistent in regard to reporting the activation of executive and inhibition control networks in abstinent AUD [42]. fMRI studies that used Go/NoGo tasks to study inhibition [36,37] have reported enhanced BOLD activation in abstinent AUD relative to healthy control participants in the fronto-striatal-parietal network [36,37] as well as in several areas involved in visual processing and cognitive and impulse control [36]. These findings were interpreted as involving a compensatory strategy for impaired cognitive processing in abstinent AUD. Similarly, although the short-term abstinent AUD group showed lower frontal brain activity compared to a control group, based on no reported difference between long-term abstinent AUD and control groups during a verbal fluency task, Dresler, Schecklmann, Ernst, Pohla, Warrings, Fischer, Polak and Fallgatter [38] suggested that an increase in frontal brain activity occurs with continued abstinence. On the other hand, Li, Luo, Yan, Bergquist and Sinha [39] reported lower activation of the right DLPFC and other cortical and subcortical brain structures during a stop signal task. Another study reported lower activation in the right DLPFC and ACC during a recognition task in abstinent individuals with AUD compared to controls [40]. Furthermore, based on a review of resting-state and event-related fMRI studies, Fein and Cardenas [41] argued that abstinence (short-term) and its maintenance (long-term) are associated with compensatory changes in synchrony so that the “top-down” executive control network has greater synchrony and the “bottom-up” stimulus-driven appetitive drive network has reduced synchrony compared to controls.

Considering the findings of the studies reviewed above, an important point to note is, however, that these findings of neural brain activation deficits or compensatory increase in activation across studies were not always associated with impaired task performance [67]. For example, although Czapla, Baeuchl, Simon, Richter, Kluge, Friederich, Mann, Herpertz and Loeber [36] reported enhanced fMRI activation in abstinent AUD participants with no concomitant impairment in the Go/NoGo task performance, their previous study, which assessed cognitive function, reported impaired response inhibition in abstinent AUD, which was a significant predictor of relapse [83]. The second important difference between studies that suggested the occurrence of a compensatory increase [36,37] and studies that suggested activation deficits [39,40], including the findings of the present study, was that the two studies that reported enhanced activation [36,37] used alcohol-related stimuli to create greater conflict in the Go/NoGo tasks. Alcohol-related stimuli have been used to study cue reactivity to examine motivational salience in alcohol-related conditions and have consistently been shown to elicit enhanced BOLD activation regardless of task conditions (see for a review [35]). On the other hand, inhibition tasks using nondrug-related stimuli have consistently shown lower activation in different functional networks during response inhibition [35].

Most studies that have used neuropsychological tests to assess inhibitory functions in abstinent individuals with AUD have also reported persistent impairments in abstinent AUD [16,17,18,19,20,21,22,23,24]. Therefore, the empirical evidence supports the occurrence of multifaceted brain impairments in addiction that are reflected even in relatively simple tasks and in the absence of any apparent behavioral response deficits. These results are largely consistent with previous neuroimaging reviews, meta-analyses [35,67,84], and the animal literature [85,86]. Therefore, in light of the discussion above, and putting the findings of neuropsychological and neuroimaging studies of response inhibition together, the proposal of an activation deficit in response inhibition impairment that persists in abstinent AUD seems plausible.

The findings of the present study tend to confirm the brain activation deficit of the response inhibition network impairment proposal and indicate low activation of the right fronto-parieto-temporal network and the ventral attentional network during response inhibition in abstinent individuals with AUD (Figure 3 and Figure 4) rather than compensatory mechanisms due to underlying deficits in response inhibition in AUD. An activation deficit during successful response inhibition in the abstinent AUD group was found despite no significant difference in behavioral performance in the NoGo trials. Moreover, the abstinent AUD group showed lower behavioral performance accuracy in the Go but not in the NoGo trials, which may appear counterintuitive at first. However, equivocal findings on behavioral measures have been reported in the extant neuroimaging literature [67] where inhibitory function brain impairments have been interpreted in the context of psychomotor slowing in AUD, even in the absence of behavioral inhibitory task performance deficits [87,88,89,90], which may partly explain the lower Go performance accuracy findings in the present study. This account of psychomotor slowing is further supported by the significantly longer average reaction times found in the abstinent AUD group compared to the control group. However, the inverse relation between age and Go performance accuracy and the positive association of age with the average reaction time also suggest an effect of the age of participants on behavioral measures, and performance may not be attributed to psychomotor slowing in AUD alone in the present sample.

Longitudinal neuroimaging designs have been used to investigate the predictive value of brain function in maintaining abstinence in addicted adults. A disengagement of the reward and executive networks during stressful cues indicates the likelihood of a relapse up to a year later in individuals with alcohol addiction, with brain measures being better predictors than behavioral measures of alcohol use and craving history [91]. Similarly, inadequate engagement of the executive and salience networks during inhibitory control has been linked to relapse in cocaine users [92,93] and cannabis users [80]. These results converge with recent reviews of neuroimaging studies in humans, implicating the reward, habit, salience, and executive networks in relapse prediction [94,95]. Importantly, as the same brain networks (except for the reward network) were also found to underlie vulnerability in youth at risk, brain function in the habit, salience, and executive networks may predict crucial transitions throughout the drug addiction cycle (onset of substance abuse, transition to addiction, and chronic relapse). These results again support the crucial underlying roles of impaired response inhibition and salience attribution in the chronic relapsing nature of the cycle of addiction.

### 4.3. Implications

Improving the understanding and characterization of inhibitory deficits and involved brain substrates in abstinent individuals with AUD is important for designing rehabilitation strategies, as evidence indicates that EFs can be improved with training, practice, and by implementing compensatory strategies at various developmental stages across the lifespan [1]. The distinction between types of inhibitory processes and their task-specific assessments has important implications. For example, in many task- and real-world scenarios, the “stopping network” is probably highly integrated with value-based judgement and mnemonic functions in other sectors of the prefrontal cortex, such as the orbital frontal, and dorsolateral sectors, in both the right and left hemispheres; however, it appears that the standard (and simple) stop signal task may not require the integrity of these other sectors. This is perhaps because it represents a relatively “pure” version of inhibitory control [26]. On the other hand, several studies have reported that training in consistent mapping of stimulus-response associations, as in modified Go/NoGo tasks, tends to improve inhibition and eventually automaticity [59,96,97,98], even if stop stimuli are not consciously perceived. Consistent with these findings, training with the modified Go/NoGo tasks rather than stop signal tasks appears to have the greatest influence on participants’ health behavior [99,100,101,102,103], as a significant reduction in weekly alcohol intake was demonstrated in a group of heavy drinkers when alcohol-related stimuli were consistently paired with the NoGo condition [100]. Similarly, the retraining of automatic action tendencies has been reported to change the alcohol approach bias in AUD patients and improve treatment outcomes a year later [104].

### 4.4. Limitations and Future Directions

Sex differences in functional brain activation patterns as well as in alcoholism are well documented in the literature. The present study involved males and, therefore, the findings of the present study may be generalized accordingly. It is, however, important to conduct similar studies in females to confirm findings. Age was not found to be associated with the first PC score $u_{1}$ that explained the variance due to a group difference. Since age was significantly different between groups, we conducted an additional analysis on an age-matched subsample (*N* = 14, abstinent AUD = 7) drawn from the sample used in the present study. The results were largely consistent with the main analysis, and the trends and directions of mean differences and associations were replicated. However, given the crucial importance of age-related brain changes in AUD, it is important that future studies account for these confounding factors and confirm the current findings with age-matched designs. Similarly, the effect of the level of educational attainment and appropriate measures of intelligence need to be controlled for in future studies to avoid possible model-fitting errors and to confirm the current findings, as there are equivocal findings on the effect of level of educational attainment on brain structure and function after controlling for IQ at age 11, e.g., [105]. While we excluded individuals who met criteria for other substance use disorders, the use of other substances in individuals with AUD who do not meet the criteria for substance use disorder is often observed as part of their clinical profile, including in the present sample. While this was beyond the scope of the present study, future studies should also examine the influence of comorbid substance use in large clinical samples. Future investigations would also benefit from an integrative approach, employing a variety of complementary technologies to analyze and understand the interconnectivity of structural, functional, and behavioral systems affected by AUD.

## 5. Conclusions

In summary, the findings of right fronto-parieto-temporal and ventral attentional network activation during response inhibition obtained in the present study seem to support the common network mechanism hypothesis. Abstinent individuals with AUD have significantly lower positive BOLD responses during response inhibition when compared to controls, mainly in the right fronto-parieto-temporal cortical regions of the brain, except for in the bilateral anterior insula. Our findings provide evidence of neural substrates of inhibitory processing deficits observed in abstinent individuals with AUD. The negative correlation of age with overall behavioral performance on the task suggests that the association may not be attributed to the effect of AUD. On the other hand, no significant correlation of age with the first PC score that captures the variance due to different levels of activation between abstinent individuals with AUD and control groups is indicative of low BOLD related activation in the fronto-parieto-temporal cortical regions that may be attributed to the persistent adverse effect of AUD.

## Figures and Tables

**Table 1:** Sociodemographic, Go/NoGo task performance, BIS-11, alcohol, and substance use profile and descriptive statistics of abstinent AUD and control participants.

	AUD			Control					
	*N* ^a^	Mean	σ	*N* ^a^	Mean	σ	*t*	*df*	*p*
Age (in years)	30	41.42	7.32	30	27.44	4.74	8.78	49.71	<0.0001
Education (in years)	30	11.93	2.35	30	15.77	1.87	−7.00	58	<0.0001
Alcohol: Age of onset (regular use ^b^)	30	15.77	2.58	12	20.50	3.80	−4.67	40	<0.0001
Alcohol: Quan/day (heavy use period) ^c^	30	11	7.66	12	3	1.60	5.58	34.57	<0.0001
Alcohol: Freq/month (heavy use period) ^c^	30	20	9.01	12	4	4.09	7.97	39.18	<0.0001
Alcohol: Quan/day (last 6 months) ^c^	30	3	6.61	18	3	1.98	0.045	46	NS
Alcohol: Freq/month (last 6 months) ^c^	30	4	8.02	18	3	3.62	0.60	43.49	NS
Length of Abstinence (in days) ^d^	30	672.93	844.94	18	57	149.76	3.89	31.97	<0.0005
Tobacco: Quan/day (last 6 months) ^c^	20	10	5.80	6	2	1.63	5.19	24	<0.0001
Tobacco: Freq/month (last 6 months) ^c^	20	28	4.83	6	14	13.82	2.47	5.37	NS
Marijuana: Freq (last 6 months) ^c^	10	99	91.38	4	19	27.61	2.58	11.74	<0.03
NoGo Performance accuracy (in %)	30	85.21	15.84	30	94.42	11.85	1.07	58	NS
Go Performance accuracy (in %)	30	91.25	9.44	30	88.83	8.03	−2.55	53.72	<0.014
Reaction Time (in milliseconds)	30	349.85	31.15	30	324.47	32.27	3.10	58	<0.003
BIS-11: Nonplanning	28	24.57	5.25	30	19.80	4.61	3.69	56	<0.001
BIS-11: Motor Impulsiveness	27	25.15	5.24	30	19.30	3.28	4.99	42.82	<0.0001
BIS-11: Attentional Impulsiveness	28	15.79	4.13	30	12.57	3.19	3.33	56	<0.002
BIS-11: Total Impulsiveness	27	65.44	11.51	30	51.67	8.60	5.15	55	<0.0001

AUD: alcohol use disorder; BIS: Barrett Impulsiveness Scale, *df*: Degrees of Freedom (Homogeneity assumed as well as not assumed based of test of homogeneity); Freq: Frequency; N: number of participants; NS: Not Significant; *p*: level of significance; Quan: Quantity; *t*: *t*-test value; σ: Standard Deviation. ^a^ Note that all participants were assessed; Table 1 includes reported data on the alcohol and substance use measures; participants who did not report data were omitted from the table for these measures. ^b^ Drink one day/per month for 6 months or more. ^c^ Quantity and Frequency mean values were rounded to the nearest whole numbers as per the reviewers’ suggestion. ^d^ Range (5–4228 days).

**Table 2:** Significant clusters identified during successful NoGo BOLD responses and their MNI coordinates, sizes in mm^3^, locations at the peak/trough, and activation directions.

Cluster No.	MNI Coordinates (R, A, S) mm	Size mm^3^	Peak/Trough Location	Activation Direction
1	(−30, 26, 0)	221	Bilateral Insula	NoGo > Go
2	(51, 19, −10)	1754	Right Inferior Frontal Gyrus	NoGo > Go
3	(52, 14, 37)	13,317	Right Middle Frontal Gyrus	NoGo > Go
4	(18, 8, 67)	174	Right Middle Frontal Gyrus	NoGo > Go
5	(5, 20, 41)	800	Right Medial Frontal Gyrus	NoGo > Go
6	(38, −61, 58)	15,599	Right Superior Parietal Lobule	NoGo > Go
7	(62, −28, −5)	656	Right Middle Temporal Gyrus	NoGo > Go
8	(52, −43, 7)	144	Right Middle Temporal Gyrus	NoGo > Go
9	(1, 61, −5)	1483	Right Medial Frontal Gyrus	Go > NoGo
10	(−1, −55, 21)	1140	Left Posterior Cingulate Gyrus	Go > NoGo
11	(−49, −71, 28)	313	Left Angular Gyrus	Go > NoGo
12	(−45, −20, 58)	289	Left Postcentral Gyrus	Go > NoGo
13	(−57, −19, 44)	102	Left Postcentral Gyrus	Go > NoGo

## Data Availability

The data presented in this study are part of the manuscript submission (Supplementary Materials).

## Supplementary Materials

The following supporting information can be downloaded at: https://www.mdpi.com/article/10.3390/bs12050121/s1.
